# Editorial: Open science to support replicability in neuroergonomic research

**DOI:** 10.3389/fnrgo.2024.1459204

**Published:** 2024-07-30

**Authors:** Klaus Gramann, Fabien Lotte, Frederic Dehais, Hasan Ayaz, Mathias Vukelić, Waldemar Karwowski, Stephen Fairclough, Anne-Marie Brouwer, Raphaëlle N. Roy

**Affiliations:** ^1^Biological Psychology and Neuroergonomics, Technische Universitaet Berlin, Berlin, Germany; ^2^Inria Center at the University of Bordeaux/LaBRI, Bordeaux, France; ^3^Fédération ENAC ISAE-SUPAERO ONERA, Université de Toulouse, Toulouse, France; ^4^School of Biomedical Engineering Science and Health Systems, Drexel University, Philadelphia, PA, United States; ^5^Fraunhofer Institute for Industrial Engineering IAO, Stuttgart, Germany; ^6^Department of Industrial Engineering and Management Systems, University of Central Florida, Orlando, FL, United States; ^7^School of Psychology, Liverpool John Moores University, Liverpool, United Kingdom; ^8^TNO Soesterberg, Radboud University Nijmegen, Nijmegen, Netherlands

**Keywords:** open access, open data, open analysis, replicability, EEG, neuroergonomics

Open access to data, code, and protocols in research is vital for accelerating scientific progress, ensuring transparency and reproducibility, and maximizing resource utilization. By making datasets and code freely available, researchers can collaborate across disciplines, verify, replicate, extend findings, and innovate more efficiently. Additionally, open data and collective use such as in open competitions (Roy et al., 2022) support education, promote equity in research opportunities, and enhance public trust and engagement. These benefits collectively advance the application of scientific principles, fostering a more inclusive and robust scientific community. However, within the neuroergonomics and neurotechnology community, this process has been remarkably slow for several reasons, resulting in a minimal number of freely available data sets with often reduced data information (Hinss et al., 2021).

Replicability and reproducibility are crucial, especially in neuroergonomics, which focuses on understanding neural processes in realistic settings that often involve resource-intensive and complex neurophysiological data collection methods like electroencephalography (EEG) or functional near-infrared spectroscopy (fNIRS). These experiments require costly and time-consuming setups, such as simulators or real vehicles. Additionally, inter- and intra-subject variabilities limit the serviceability of datasets. This Research Topic (RT) thus invited submissions that support data sharing of such complex and expensive (neuro-)physiological data collected in naturalistic or real-life conditions, and that investigate different aspects of the user interacting with technical systems. Besides sharing data sets, authors were encouraged to provide their signal processing code using open-source software/platforms and to follow the guidelines from the Organization for Human Brain Mapping (OHBM) and the Brain Imaging Data Structure (BIDS) (Pernet et al., 2019) for data labeling and organization.

This RT accepted four submissions providing valuable multimodal data that can be used by the community to replicate and extend the reported analyses. The first data set contributed by Kueper et al. provides 64-channel EEG and 8-channel EMG data of 8 healthy participants interacting with an exoskeleton. The original study in which the data was collected investigated whether tactile detection of incorrect behavior in an exoskeleton or orthosis evokes similar event-related potentials (ERPs) as visually observed incorrect behavior to use the resultant information to correct the behavior. To this end, the authors introduced easily recognizable force direction errors, opposing the planned body movements, and recorded behavioral, EEG, and EMG data during normal and erroneous interactions with the exoskeleton. It should be noted that this data set was used for an IJCAI 2023 competition entitled: “IntEr-HRI Competition: Intrinsic Error Evaluation during Human-Robot Interaction,” hence fostering the ideas of several research teams to further the development of passive BCIs and adaptive Human-Robot Interaction (HRI).

The second contribution, by Pan et al., provides an extensive data analysis pipeline based on the data published by Kueper et al.. The pipeline received a prize as the winning approach of the above-mentioned competition. The authors focus on developing an automated pipeline for online error detection in an assistive device for improving the reliability of orthosis movements in real-time. This approach makes two major contributions to the field, the first demonstrating the feasibility of integrating two temporal derivative features with an effect size-based feature selection strategy, specifically for online EEG-based BCIs. The second contribution is the introduction of an innovative approach for continuous online error prediction, incorporating a simple noise rejection technique to reduce false alarms. The methodology allows for continuous error detection, with the potential to significantly advance practical applications in neuroadaptive technology and HRI.

Gehrke et al. contribute with a dataset that addresses one of the key challenges in designing immersive virtual reality (VR) experiences: creating an environment that closely mimics the real world to enhance users' sense of presence. The available Mobile Brain/Body Imaging data (Gramann et al., 2011; Jungnickel et al., 2018) in Motion-BIDS format (Jeung et al., 2024) includes brain recordings via 64-channel EEG together with behavioral responses, and motion capture during an interactive VR experience in which participants were asked to point to a virtual object that appeared at different locations in front of them (see Gehrke et al., 2019, 2022). In three different sensory conditions, only visual, visual plus tactile, or visual plus tactile combined with force feedback are provided. When touching the virtual object sensory feedback about the successful touch would be provided (visual only; visual plus tactile; visual, tactile, and force feedback) indicating the completion of the trial. In some trials, the feedback was provided prematurely, creating an error in the interaction. By violating the users' predictions about the VR's interaction, prediction errors were recorded and analyzed providing a multimodal prediction error data set that allows for investigating user presence in the virtual scenario.

Finally, Demirezen et al.'s systematic literature review summarizes the current efforts toward reproducibility in both machine learning and EEG research related to mental workload. They formulate guidelines structured around the Cross-Industry Standard Process for Data Mining (CRISP-DM) framework, aiming to ensure methodological transparency and comprehensiveness while enhancing collaboration, knowledge-sharing, and the reliability of EEG and machine learning techniques. The authors further extracted machine learning studies using EEG to estimate mental workload and assessed the reproducibility of these studies using the previously described guidelines, highlighting both studied and overlooked areas and identifying current challenges. Consistent with the topic of the RT, one of the identified challenges was the lack of open sharing of data and code.

The limited number of published data sets, open analysis pipelines, and guidelines for reproducible research in neuroergonomics are highlighted in this Research Topic marking an essential initial step toward enhancing open-science practices in the field. This RT aims to encourage the sharing of complex and costly neurophysiological data collected under naturalistic conditions, and robust analysis pipelines, in order to facilitate replication, validation, and extension of studies. These efforts not only foster a collaborative and transparent scientific community but also pave the way for faster translation of neuroergonomics toward practical applications. As more researchers adopt these open-science approaches, the potential for groundbreaking advancements and everyday usability in neuroergonomics will significantly increase, ultimately driving the field toward more inclusive and impactful scientific progress.

## Author contributions

KG: Conceptualization, Writing – original draft, Writing – review & editing. FL: Conceptualization, Writing – review & editing. FD: Conceptualization, Writing – review & editing. HA: Conceptualization, Writing – review & editing. MV: Conceptualization, Writing – review & editing. WK: Conceptualization, Writing – review & editing. SF: Conceptualization, Writing – review & editing. A-MB: Conceptualization, Writing – review & editing. RR: Conceptualization, Writing – review & editing.
